# Extracellular influences on tumour angiogenesis in the aged host

**DOI:** 10.1038/sj.bjc.6604144

**Published:** 2008-01-08

**Authors:** C C Sprenger, S R Plymate, M J Reed

**Affiliations:** 1 Molecular and Cellular Biology Program, University of Washington, 1959 NE Pacific, Seattle, WA 98195, USA; 2 Division of Gerontology, Department of Medicine, University of Washington, Harborview Medical Center, 325 9th Avenue, Box 359625, Seattle, WA 98104, USA; 3 Veterans Affairs Puget Sound Health Care System, Seattle Division, 1660 South Columbian Way, Seattle, WA 98108, USA

**Keywords:** prostate cancer, microenvironment, senescence, angiogenesis, growth factors, matrix metalloproteinases

## Abstract

Whether tumours are epithelial or non-epithelial in origin, it is generally accepted that once they reach a certain size all solid tumours are dependent upon a vascular supply to provide nutrients. Accordingly, there is great interest in how the extracellular environment enhances or inhibits vascular growth. In this minireview, we will examine key extracellular components, their changes with ageing, and discuss how these alterations may influence the subsequent development of tumour vasculature in the aged host. Because of the tight correlation between advanced age and development of prostate cancer, we will use prostate cancer as the model throughout this review.

During an organism's lifespan, almost every aspect of its phenotype will undergo alterations, including the components of the extracellular environment. It is increasingly evident that there is a dynamic interaction between the molecules of the extracellular space and the surrounding cells. The architecture of the extracellular space is important for maintaining proper cellular function; loss of tissue architecture is a defining characteristic of epithelial cancers. A microenvironment that provides the correct cues can serve as a powerful tumour suppressor and can even revert cells containing preneoplastic as well as oncogenic mutations back to a normal phenotype (Bissell and Radisky, 2001; Campisi, 2005; Nelson and Bissell, 2006). The processes of living and ageing, however, continually alter the composition, and thus the architecture, of the extracellular space.

Traditionally, interactions between tumour cells and various growth factors have been the focus in cancer. However, there is increasing interest in the roles of other proteins in the extracellular environment on tumour progression. The term ‘microenvironment’ now includes the extracellular matrix (collagens, laminins, matricellular proteins) and soluble factors (hormones, cytokines, growth factors, enzymes) that are released by resident and circulating cells or secreted by other organs.

All non-circulating cells are physically linked to the extracellular space via cell membrane receptors such as integrins and syndecans. Signalling through these receptors influences changes to the cell's cytoskeleton network, which is connected to the nuclear matrix and chromatin. Alterations in the cytoskeleton modify gene expression, which in turn leads to changes in the chemical and protein composition of the microenvironment (Nelson and Bissell, 2006). In epithelial cancers, transformed epithelial cells, reactive stroma, recruited blood vessels, and infiltrating macrophages, lymphocytes, and leukocytes also contribute to the microenvironment (Nelson and Bissell, 2006; Tan and Coussens, 2007). In this review, we will use prostate cancer as the model and focus on the potential roles of extracellular matrix proteins and soluble factors during tumour angiogenesis in the aged host (Figure 1).

## EPITHELIAL CANCERS

Age-associated epithelial cancers, such as breast and prostate cancer, contribute significantly to mortality in the elderly. One possible mechanism by which the body defends itself against epithelial cancers is to halt replication of damaged cells by senescence, in which the cells are replicatively arrested but still metabolically active. Since somatic mutations are believed to accumulate throughout life, senescence is important in preventing the formation of epithelial tumours in the young. Accumulation of mutations alone, however, is not sufficient to cause cancer. Currently, one view is that these ‘initiated’ cells require a permissive microenvironment in which to progress (Campisi, 2005; Nelson and Bissell, 2006). The accruement of senescent cells may provide such an environment due to factors secreted by these cells that compromise tissue structure and function. Once a cell has entered senescence, its transcriptome is altered such that genes associated with angiogenesis are activated. Inflammatory cytokines (IL-8), growth factors (TGF-*β*, EGF), matrix metalloproteinases (MMPs), and extracellular matrix proteins (laminins, collagens, fibronectin) are among the genes upregulated by senescent cells (Zhang *et al*, 2003; Campisi, 2005; Bavik *et al*, 2006). This alteration in expressed genes affects not only the senescent cell itself, but the cells surrounding it as well. Senescent fibroblasts that were co-cultured with breast or prostate epithelial cells increased the proliferation and tumorigenicity of those epithelial cells, both *in vitro* and *in vivo* (Campisi, 2005; Bavik *et al*, 2006). Senescence, then, inhibits cancer formation early on but over time the build up of senescent cells alters the microenvironment to one that can promote the initiation of epithelial cancers.

Clinical observations suggest that while ageing confers the greatest risk of developing cancer, once initiated, histologically similar tumours behave less aggressively in the aged individual (Ershler, 1986). This premise was further supported by animal studies in which young and aged mice received identical inocula of tumour cells and were subsequently monitored for tumour growth and aggressiveness (Kreisle *et al*, 1990; Pili *et al*, 1994). Proposed mechanisms for differences in tumour behaviour in young *vs* aged hosts have focused on age-related deficits in immune-mediated responses that directly and indirectly promote tumour growth (such as a lack of inflammatory cells and their associated cytokines), increases in apoptosis, and decreases in pathological angiogenesis (Ershler, 1986; Kreisle *et al*, 1990; Pili *et al*, 1994). The aged microenvironment, it has been argued, is less permissive to pathological angiogenesis and tumour growth than the milieu of tissues found in the young. Such an alteration in tissue architecture has been thought to be an adaptive response to the greater risk of cancer conferred by senescence and environmentally induced changes in the epithelial and stromal cells (Campisi, 2005). More recently, however, we have shown that prostate epithelial tumours can provide a microenvironment that allows certain tumours to grow equally well in aged and young mice (Reed *et al*, 2007).

The aged microenvironment, likewise, affects the metastasis of the primary tumour. Recent data from Kaplan *et al* (2006) suggest that tumour metastases are determined by preparation of a bone marrow-derived ‘metastatic niche’ prior to the arrival of cancer cells (Kaplan *et al*, 2006). When aged marrow was used to replace the marrow in young irradiated mice, tumour metastases decreased. Conversely, when marrow from young donors was used to replace marrow in older irradiated mice, metastases increased. Langley and Fidler (2007) further reviewed the myriad of factors involved in metastases, including the angiogenic component, and concluded that interactions of the tumour cells with the host homoeostatic mechanisms are highly variable and complex (Langley and Fidler, 2007). Therefore, the reasons behind an apparent decrease in metastases in the aged host *vs* young host are many fold and likely vary with the type of cancer. In this minireview, we will focus on how age-induced modifications of the microenvironment influence angiogenesis of the primary tumours.

## EXTRACELLULAR MATRIX

The specific effects of age on the extracellular matrix have not been well delineated within the tumour microenvironment. However, studies of age-related alterations of the matrix in other tissues, and of changes in tumour matrix in non-aged hosts, provide a basis for discussion of collagen and laminin, the best studied of these proteins.

Collagen I is a heterotrimeric, fibrillar protein that is the major structural extracellular protein in most tissues (Chung *et al*, 2005). Collagen I has been the most extensively examined collagen in aged hosts and the consensus is that ageing confers a progressive decrease in collagen I synthesis at the same time there is an increase in collagen I degradation. There are important exceptions to this premise, such as the increased collagen I deposition that is often noted in aged hearts (Gazoti Debessa *et al*, 2001). Although the cardiac changes are primarily a response to injury or hypertension, and not ageing *per se*, the observations with respect to collagen I underscore the need to use the term ‘deregulation’ to describe many of the changes in the matrix in aged organs.

Studies examining mechanisms of decreased collagen I content in aged tissues have noted that lower levels of fibrogenic growth factors, such as transforming growth factor-*β* (Ashcroft *et al*, 1997), contribute to less collagen I synthesis and subsequent scarring. At the same time, elevated matrix metalloproteinase activity mediates increased collagen I degradation. Whether the latter results from an increase in collagenase and other MMPs or a decrease in tissue inhibitors of MMPs is still a matter of debate, but the end result is a looser, less organized collagen network (Hornebeck *et al*, 2002). Whereas some have suggested that a less dense collagen matrix facilitates vascular in-growth (Reed *et al*, 2005), studies of angiogenesis in most organs have demonstrated decreased capillary density with age (Rivard *et al*, 1999). Alterations in collagen I that have functional consequences include age-related deficits in integrin-collagen binding that result in less robust cell adhesion and migration (Reed *et al*, 2001), which could contribute to delayed tissue repair.

Although diminished collagen I content results in less scarring and fibrosis in most aged tissues (with the exception of the heart as noted above), the implications for tumour angiogenesis and growth are largely a matter of conjecture and depend on the tumour cell type. For example, we found that the amount of collagen I in melanomas from aged mice was decreased compared to levels found in prostate tumours from aged mice; likewise, we found decreased vessel density in these melanomas compared to the prostate tumours (Reed *et al*, 2007). The therapeutic implications are especially of interest in the treatment of cancers that produce large quantities of collagen such as prostate. One could surmise that if a cancer cell can be modified to secrete less collagen, there will be decreased support for vascular in-growth and subsequent tumour progression.

While collagen I is the best-studied extracellular matrix protein in non-tumour aged tissues, the examination of laminins in tumour angiogenesis has been restricted to non-aged hosts. Laminins (LM) are large matrix glycoproteins composed of highly homologous *α*, *β*, and *γ* chains and are the main constituent of basal membranes (a special matrix that separates different cell types from one another, such as endothelial or epithelial cells from the surrounding stroma). Laminins are crucial components of the tissue architecture, as well as modulators of cell behaviour (Patarroyo *et al*, 2002). The laminin *α*4 chain is expressed by most endothelial cells throughout the body and plays an important role in post-developmental angiogenesis associated with inflammation and tumours (Zhou *et al*, 2004). LM411 (*α*4*β*1*γ*1) (formerly known as laminin-8) facilitates endothelial proliferation and protects endothelial cells from apoptosis (DeHahn *et al*, 2004). Sprouting and tumour blood vessels express LM411, whereas normal blood vessel maturation and loss of malignant characteristics are associated with conversion to LM421 (*α*4*β*2*γ*1) (formerly known as laminin-9) (Zhou *et al*, 2004). LM411 facilitates tumour progression, but the presence of LM421 may be equally important in preventing metastases, as *lama4*^−/−^ mice exhibit uncontrolled blood vessel proliferation following injury and have increased tumour growth and metastasis (DeHahn *et al*, 2004; Zhou *et al*, 2004).

Senescent stromal cells highly secrete laminins. Accordingly, laminins have been postulated to influence the cancer phenotype of breast and prostate epithelium. Recent studies have shown that senescent prostate epithelial cells found in regions of benign prostatic hyperplasia as well as senescent prostate fibroblasts have increased expression of the laminin *α*4 and *β*1 chains (Luo *et al*, 2002; Bavik *et al*, 2006). Fujita *et al* (2005) also demonstrated a switch from LM421 to LM411 expression in breast tumour vasculature, implying that increased expression of the LM411 chains may be associated with progression of some epithelial cancers (Fujita *et al*, 2005). This group recently reported regression of glioblastoma tumours in mice following administration of LM411 inhibitors (Fujita *et al*, 2006). It would be of interest to examine if a similar outcome occurred in mouse models of breast or prostate cancer since aberrant expression of LM411 by senescent and epithelial tumour cells appears to influence the angiogenic potential of adjacent endothelial cells. Thus, in the aged host, accumulation of senescent cells may facilitate epithelial tumour growth, in part, via increased expression of laminins associated with tumour angiogenesis.

## MATRICELLULAR PROTEINS

The term matricellular refers to proteins of the extracellular space that regulate cell function without providing significant structural support. Although the size of the family of molecules designated as ‘matricellular’ continues to grow, only a few member proteins have been examined in aged hosts. Key molecules investigated in ageing and in tumour biology include thrombospondin 1 (TSP1) and secreted protein acidic and rich in cystein (SPARC).

Thrombospondin 1 is a large heterotrimer whose expression increases in most aged cells and tissues (Naumov *et al*, 2006). The negative effects of TSP1 on endothelial cell function have resulted in great interest in this protein in tumour angiogenesis and progression. In many cancers, TSP1's presence is associated with a non-angiogenic phenotype and tumour regression; the absence of TSP1 expression is correlated with an angiogenic switch and metastases (Naumov *et al*, 2006). Thrombospondin 1 inhibits angiogenesis by blocking growth factor-mediated angiogenic functions such as proliferation and migration as well as by enhancing apoptosis in activated endothelial cells (Colombel *et al*, 2005).

In the prostate, androgens repress the transcription of TSP1. However, it is now understood that the clinical implications of TSP1 expression on tumour vascularity and growth depend on the duration of exposure. Androgen withdrawal initially leads to increases in TSP1 and vessel regression; however, with continued exposure prostate cancer angiogenesis and growth continue despite persistently high levels of TSP1 (Colombel *et al*, 2005). Similar results have been reported in breast cancers: persistently high levels of TSP1 in the tumour stroma ultimately result in disease progression, an effect that may result from increased expression of VEGF (Fontana *et al*, 2005). These conflicting effects have dampened enthusiasm for the use of fragments of TSP1 in clinical intervention studies.

Secreted protein acidic and rich in cystein is a secreted glycoprotein that is highly expressed in injured and inflamed tissues. Accordingly, high levels of SPARC are found in many aged organs and in numerous cancers (Framson and Sage, 2004). Intact SPARC inhibits the angiogenic response by impairing proper collagen alignment and blocking pro-angiogenic growth factors (Kupprion *et al*, 1998). At the same time it has been reported that cleaved SPARC might facilitate vessel growth by enhancing endothelial cell proliferation (Sage *et al*, 2003). In aged tissues, the complexities surrounding the role of SPARC in the angiogenic response are obviated by age-related deficits in the levels of growth factors and other pro-angiogenic mediators (Reed *et al*, 2005). Nevertheless, the relative ease by which *in vivo* SPARC expression can be manipulated has resulted in continued enthusiasm for its therapeutic potential in highly vascularized tumours (Elola *et al*, 2007).

## ANDROGENS

Although serum androgen levels decrease with age, levels of active androgen in the prostate, dihydrotestosterone, do not decrease due to increased activity of the 5*α*-reductase enzyme, which converts testosterone (T) to dihydrotestosterone (Bonnet *et al*, 1993). In malignant prostate epithelium androgens can stimulate angiogenesis (Colombel *et al*, 2005). Following androgen withdrawal in androgen-dependent tumours, there is a decrease in angiogenesis associated with tumour regression. However, there is invariably a return of tumour that is castration resistant. These tumours are commonly referred to as androgen-independent (AI), although castration-resistant may be a more appropriate term since they still contain significant levels of T and dihydrotestosterone (Mostaghel *et al*, 2007). In castration-resistant tumours, there is an increase in angiogenesis that is associated with an increase in MMP-9 (London *et al*, 2003). These studies indicate that tumour angiogenesis in prostate cancer is associated first with androgens, then with an increase in matrix remodelling proteases. Further, these data imply that anti-angiogenic drugs should be of potential therapeutic benefit. However, no definitive clinical trials have been published, which may reflect a unique ability of prostate cancer to bypass the usual age-associated inhibition of angiogenesis.

## GROWTH FACTORS

Although many growth factors regulate the angiogenic response in the tumour microenvironment, we will focus our discussion on vascular endothelial growth factor (VEGF) and insulin-like growth factor-1 (IGF-1). These two traditional growth factors directly facilitate endothelial cell functions that promote blood vessel formation and have been examined in prostate cancer progression. Other mediators, such as IL-8 and associated inflammatory cytokines that modulate endothelial cell behaviour, will not be discussed due to their intricate relationship with the immune system of the host during tumour initiation and growth.

It is generally accepted that ageing compromises the ability of cells to produce angiogenic growth factors, including VEGF (Rivard *et al*, 1999). Vascular endothelial growth factor is the most potent of the numerous mediators that induce endothelial cell functions and facilitate new vessel formation. The primary stimulus for VEGF synthesis is hypoxia. However, the response to low oxygen tension is blunted in aged tissues as a result of defects in hypoxia-inducible factor 1 (HIF-1), the transcription factor responsible for VEGF synthesis (Rivard *et al*, 2000). Although one would predict that decreased VEGF expression would confer an element of protection against tumour vascularisation and subsequent growth in aged hosts, the clinical data supporting this premise are lacking. In prostate cancer, while VEGF levels are not predictive of positive biopsy results (Peyromaure *et al*, 2005) higher plasma levels of VEGF are associated with metastases and a poorer prognosis (Duque *et al*, 1999). Using a transgenic mouse model of prostate cancer, Isayeva *et al* (2007) demonstrated that inhibitors of the VEGF2 receptor delayed tumour progression only when administered in the early stages of prostate cancer, before a significant rise in VEGF levels was observed. This same inhibitor was ineffective if administered during the later stages of prostate cancer, when VEGF levels were high (Isayeva *et al*, 2007). Thus, the minimal effects on tumour progression in clinical trials of angiogenesis inhibitors may be due to the advanced stage of prostate cancer being targeted. It is, therefore, likely that the decrease in VEGF seen in many tissues with ageing does not inhibit the development of prostate cancers in aged men. Administration of angiogenesis inhibitors may be more effective if given earlier in the course of prostate cancer progression, prior to a rise in VEGF levels, or in conjunction with other therapeutic interventions such as androgen ablation.

Insulin-like growth factor-1 is a potent stimulator of cellular proliferation and survival as well as tumour growth. While serum levels of IGF decrease with age, within the aged population those individuals with the highest levels of serum IGF-1 have the greatest risk of developing epithelial cancers such as prostate cancer (Kaplan *et al*, 1999). During progression of prostate cancer, local levels of both IGF-1 and its receptor (IGF-1R) increase (Kaplan *et al*, 1999). Like many growth factors, IGF-1 has the potential to reverse the age-associated decline in endothelial cell function (Thum *et al*, 2007). Moreover, IGF-1 upregulates the expression of modulators of endothelial cell function such as VEGF, MT1-MMP, and MMP2; this regulation requires signalling through the IGF-1 receptor via both the PI3 kinase and MAP kinase pathways (Miele *et al*, 2000; Grzmil *et al*, 2004; Zhang *et al*, 2004). Goel *et al* (2004) reported that interactions between the IGF-1R and *β*_1_ integrins also activated signalling through both the PI3 kinase and MAP kinase pathways, which resulted in enhanced prostate tumour cell migration on and invasion through the extracellular matrix (Goel *et al*, 2004). Although tumour angiogenesis has been associated with increased expression of *β*_1_ integrins and increased signalling through the PI3K pathway (Stupack and Cheresh, 2002), the direct effect of IGF-1R-integrin *β*_1_ interactions on vessel growth in cancers has not been studied. Accordingly it is implied, but not proven, that increased levels of IGF-1 in the aged prostate could promote endothelial cell function thereby resulting in similar levels of vascularisation, and primary tumour growth, in young and old hosts.

## MATRIX METALLOPROTEINASES

The extracellular environment contains numerous classes of enzymes that regulate the controlled degradation of matrix proteins. Many of these proteases also modulate other cellular functions, either directly by interacting with receptors at the cell surface or indirectly by activation of latent molecules in the extracellular milieu. Within the context of tumour progression and angiogenesis, the matrix metalloproteinases (MMPs) are the most widely studied class of molecules. Although it is a widely held belief that tissue levels of MMPs increase with ageing, more recent studies indicate that changes in MMP activity in most aged organs reflect a deregulation rather than pervasive increases (Reed *et al*, 2000). Accordingly, some aged tissues show decreased matrix turnover at the same time others demonstrate increased MMP activity.

During cancer progression, organ and tumour cell-specific changes in MMPs, rather than host age, determine the influence of matrix degradative enzymes on subsequent tumour growth. The lack of a specific and pervasive age effect on MMP levels is important clinically as it is generally accepted that the ability of solid tumours to express gelatinases is positively correlated with their invasive potential and subsequent poor clinical outcomes (Mancini and Di Battista, 2006). Prostate tumours, in particular, express increasing amounts of MMP2 and MMP9 as they progress to higher-grade tumours and greater degrees of metastatic potential (Wood *et al*, 1997). The influence of MMPs on tumour propagation results from both direct and indirect mechanisms (Mancini and Di Battista, 2006). Direct effects, via degradation of the matrix, result in a more permissive environment for cell migration and invasion. The subsequent facilitation of the angiogenic response results in a greater blood supply to the tumour. Indirect effects of MMP activity include activation of other pro-MMPs, cleavage of regulatory precursor molecules at the cell surface, and induction of nascent chemokines and growth factors that require enzymatic activation.

We have shown in aged animals that angiogenesis and tumour growth are inhibited in some solid tumours, such as melanomas, but in prostate cancer equivalent angiogenesis and tumour growth occurred in both young and old animals. Moreover, the prostate tumours had high levels of gelatinase (MMP2 and MMP9) expression and activity (Reed *et al*, 2007). The relationship between a well-formed matrix and MMP expression is expected as extracellular matrix proteins regulate, in part, the production of the enzymes responsible for their turnover and degradation (Phillips and Bonassar, 2005). Once tumour cells express MMPs, they can induce MMP secretion from their associated stromal cells thereby further amplifying their potency (Stuelten *et al*, 2005). MMP activity also has been shown to be a key component of VEGF-induced angiogenesis in tumours (Bergers *et al*, 2000), reflecting another pathway by which MMPs interact with components of the ECM to facilitate vessel formation and tumour growth. Although the modulation of MMPs has resulted in minimal effects in the therapeutic arena, it is notable that these studies employed general MMP inhibitors. The development of more specific and potent MMP inhibitors, in conjunction with other interventions, may result in improved clinical efficacy.

## CONCLUSION

Location, location, location: body-wide levels of factors associated with angiogenesis may decrease with ageing, but their level of expression can increase locally. The prostate provides a unique model for this paradigm. For example, while serum levels of IGF-1 decrease with age, prostatic levels increase during prostate cancer progression. This local increase in IGF-1 leads to an upregulation in factors, such as VEGF, MT1-MMP, and MMP2, which modulate endothelial cell function and subsequent angiogenesis. Furthermore, senescent cells are more prevalent with host age and display a transcriptome that parallels angiogenesis. Growth factors, cytokines, MMPs, collagens, laminins, and integrins are all upregulated by senescent cells. Senescent fibroblasts and epithelial cells may subsequently alter the local microenvironment to one that promotes angiogenesis and epithelial tumour growth. Consequently, while angiogenesis is generally impaired in aged tissues, the local microenvironment of primary epithelial tumours in the aged host may be as supportive of angiogenesis as that found in the young.

## Figures and Tables

**Figure 1:**
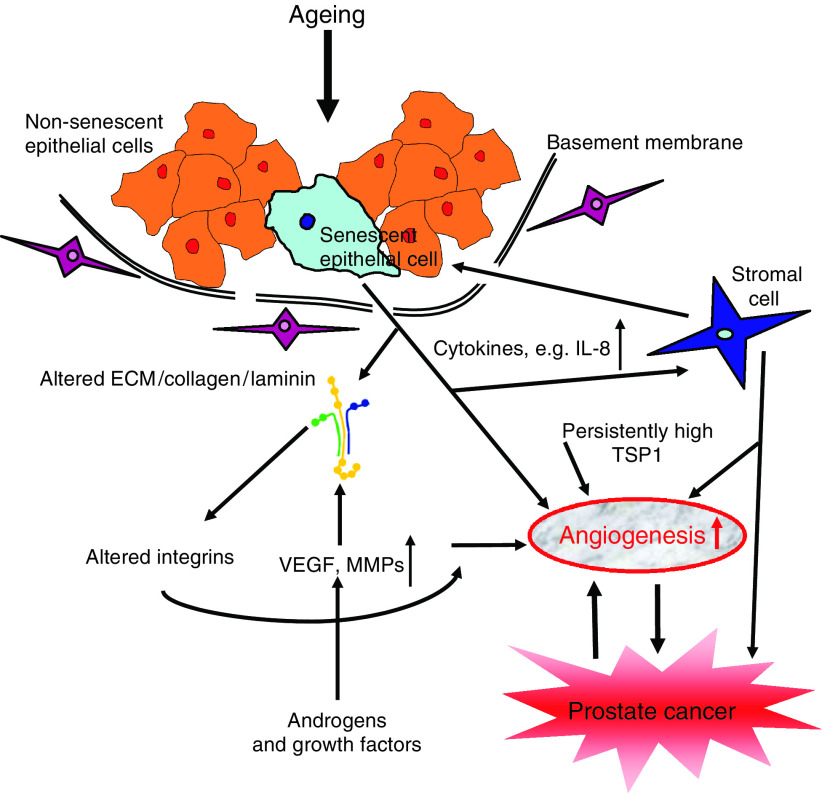
Effects of the aging microenvironment on angiogenesis of prostate tumours. The presence of senescent cells increases with age. These cells alter their expression of ECM proteins, which in turns modifies the composition of the microenvironment. Although the stromal cell has not been shown to senesce in the prostate, the cytokines produced by senescent epithelium influence stromal cell function and secretions. Local increases in hormones, growth factors (such as IGF-1), and matricellular proteins such as thrombospondin (TSP1) further alter the microenvironment. The aged prostate microenvironment, therefore, contains many components that are pro-angiogenic, thus supporting the growth of transformed epithelial cells and enhancing angiogenesis of the primary tumour in the aged host.
